# mTOR-inhibiting pharmacotherapy for the treatment of non-infectious uveitis: a systematic review protocol

**DOI:** 10.1186/s13643-018-0745-2

**Published:** 2018-06-09

**Authors:** Joshua Blair, Robert Barry, Philip I. Murray, David J. Moore, Alastair K. Denniston

**Affiliations:** 1 Department of Ophthalmology, University Hospitals Birmingham NHS Foundation Trust, Birmingham, UK; 2 Centre for Rare Diseases, Institute of Translational Medicine, Birmingham Health Partners, Birmingham, UK; 3 Academic Unit of Ophthalmology, Institute of Inflammation and Ageing, University of Birmingham, Birmingham, UK; 4 Birmingham and Midland Eye Centre, Sandwell & West Birmingham NHS Trust, Birmingham, UK; 5 Institute of Applied Health Research, University of Birmingham, Birmingham, UK; 6 NIHR Biomedical Research Centre for Ophthalmology, Moorfields Eye Hospital NHS Foundation Trust and UCL Institute of Ophthalmology, London, UK

**Keywords:** Uveitis, mTOR inhibitors, Mammalian target of rapamycin, Immunosuppression, Systematic review

## Abstract

**Background:**

Non-infectious uveitis represents a sub-type of intraocular inflammation often associated with disorders of immune dysregulation. If untreated, the intraocular inflammation may progress to severe visual impairment and blindness. Current treatment is heavily reliant on systemic corticosteroid, often at doses associated with severe side effects. There is a need for efficacious corticosteroid-sparing immunomodulatory therapy for these patients. Current immunomodulators include various immunosuppressants and biologics but mammalian target of rapamycin (mTOR) inhibitors (such as sirolimus and everolimus) may also be contenders for this role.

The systematic review proposed here will evaluate the evidence for the use of sirolimus and everolimus in the context of non-infectious uveitis.

**Method/design:**

Standard systematic review methodology will be used to identify, select and extract data from any comparative or non-comparative study of mTOR inhibitors in patients with non-infectious uveitis excluding case reports. Searches of bibliographic databases (MEDLINE, EMBASE, The Cochrane Library and CINAHL) and clinical trials registers will be performed, with no restriction on language or date of publication. Translation of non-English language articles will be undertaken where necessary.

The primary outcome of interest will be uveitis activity as measured by vitreous haze. Secondary outcomes will include other pre-specified measures of uveitis activity (such as anterior chamber cells or central macular thickness) best corrected visual acuity, heath-related quality of life, requirement for concurrent treatment and adverse events.

Risk of bias assessment will be performed appropriate to each study design. Study selection, data extraction and risk of bias assessment will be undertaken by two reviewers independently. Data will be grouped, tabulated and narratively synthesised. Meta-analysis will be undertaken where appropriate clinical and methodological homogeneity exists. The review will be published according to PRISMA guidance.

**Discussion:**

Studies of various designs have investigated the clinical use of mTOR inhibitors for non-infectious uveitis, and a large international randomised controlled trail of sirolimus for non-infectious uveitis is due to report. The findings of this systematic review will help inform ophthalmologists and aid the improvement of treatment protocols for non-infectious uveitis with regard to the use of mTOR inhibitors.

**Systematic review registration:**

PROSPERO CRD42017056390

**Electronic supplementary material:**

The online version of this article (10.1186/s13643-018-0745-2) contains supplementary material, which is available to authorized users.

## Background

Uveitis is a serious and potentially blinding condition and is a significant cause of legally recognised blindness in the working-age population in many parts of the world [1–3]. Most cases of non-infectious uveitis appear to be autoimmune in nature, occurring in either isolation (termed ‘idiopathic uveitis’) or in association with systemic conditions featuring immune dysregulation (e.g. sarcoidosis, Behcet’s disease) [2, 4, 5].

First-line treatment usually involves immunosuppression with corticosteroids, which can be delivered by topical, local (intraocular injections or implants) or systemic (often oral prednisolone) routes [6]. Corticosteroids achieve rapid and effective control of uveitis, but are limited by development of severe side effects, which become particularly prevalent with increasing doses and prolonged use [7]. Safe maintenance doses of corticosteroids are generally considered to be less than 10 mg of prednisolone orally per day [8].

Patients requiring high doses of corticosteroids, and those in whom disease is not adequately controlled by corticosteroids, require the addition of second-line treatments [8]. Treatment options include mycophenolate mofetil, azathioprine and calcineurin inhibitors (such as tacrolimus and ciclosporin); however, these agents lack the speed of onset and efficacy of corticosteroids and many are associated with the development of different, but equally limiting side effects [5]. Multiple second-line agents may be used in combination, and it is common to switch between classes to achieve the desired therapeutic effect. Biologic therapies and cytotoxic alkylating agents may also be used in severe or recalcitrant disease [5, 8]. There remains a clear need for efficacious steroid-sparing immunomodulatory therapy for patients with non-infectious uveitis, aiming to achieve uveitis control at a cost of the fewest possible side effects [5].

Mammalian target of rapamycin (mTOR) inhibitors have recently emerged as an area of interest in ophthalmology and may be contenders for this role. mTOR inhibitors are a class of immunomodulatory agents which mediate their anti-inflammatory effects through inhibition of T cell function. The class includes sirolimus (also known as rapamycin) and everolimus [5, 8]. These agents inhibit the action of mTOR, which is a serine/threonine kinase with effects on many cell processes [9]. In the context of T cells, the mTOR inhibitors interfere with signal transduction downstream of the cytokine receptor for IL-2, preventing IL-2 from causing T cell proliferation and differentiation [10, 11]. This may have a beneficial effect in the context of uveitis, as the immune dysfunction in non-infectious uveitis is thought to be primarily T cell mediated [2, 11].

A number of studies have investigated mTOR inhibitors in non-infectious uveitis. A scoping search of the Cochrane Library and the international prospective register of systematic reviews (PROSPERO) has revealed no published or ongoing systematic reviews. It is therefore timely to perform a systematic review to evaluate the evidence for the use of sirolimus and everolimus in the context of non-infectious uveitis.

## Methods/design

### Aim

The aim of the review is to assess the effectiveness and safety of the mTOR inhibitors sirolimus and everolimus for the treatment of non-infectious uveitis. This will be achieved by conducting a systematic review of studies which:

- Evaluate or describe the use of an mTOR inhibitor (sirolimus or everolimus) in the context of human non-infectious uveitis
- Compare an mTOR inhibitor to a non-pharmacological or pharmacological treatment

Standard protocol-guided systematic review methods will be used.

This systematic review protocol has been registered in the international prospective register of systematic reviews (PROSPERO) database (ref. CRD42017056390) and reported according to Preferred Reporting Items for Systematic Reviews and Meta-Analyses Protocols (PRISMA-P) guidelines (see Additional file 1).

### Searches

The following sources will be searched for literature to review:

- Bibliographic databases of published studies
  - ○ MEDLINE (Ovid)
  - ○ MEDLINE in process
  - ○ EMBASE (Ovid)
  - ○ CINAHL (EBSCO)
  - ○ The Cochrane Library (CENTRAL Register of Controlled Trials)
- Registers of clinical trials
  - ○ WHO International Clinical Trials Registry Platform (ICTRP) portal (www.who.int/ictrp) which comprises a portal to multiple registers including Clinicaltrials.gov, the European Clinical Trials Database (EudraCT; www.clinicaltrialsregister.eu) and the International Standard Randomised Controlled Trials Number database (ISRCTN; www.controlled-trials.com)
- Abstract and Conference Proceedings
  - ○ British Library ZETOC
  - ○ Conference Proceedings Citation Index (Web of Science)
- Grey Literature
  - ○ OpenGrey (www.opengrey.eu)

For bibliographic databases, the search strategy will combine index and free text terms for the mTOR inhibitors both as a class and as individual drugs (such as sirolimus and everolimus), and the condition (non-infectious uveitis). A sample search strategy for MEDLINE is provided in Appendix 1, which will be adapted for use in each database.

The other sources listed above will be searched in a more iterative way, where a complex search strategy cannot be used. There will be no restriction on the language or year of publication. Search results will be entered into an EndNote (Thomson Reuters) database and duplicate entries removed.

### Selection criteria

The following criteria will be used to select literature for review:

- Study design
  - ○ Any study design will be included excluding single case reports.
- Participants
  - ○ Participants of any age, gender or ethnicity with non-infectious uveitis
- Intervention and comparator
  - ○ Intervention: an mTOR inhibitor
  - ○ Comparator (where present): Any comparator, for example this may be an mTOR inhibitor, another pharmacological agent, or the use of no agent or placebo.
- Outcomes
  - ○ Outcomes will not be used to select studies. Outcomes important for the aims of this review are:
  - ○ Primary outcome: clinical assessment of uveitis activity by vitreous haze score [4]
  - ○ Secondary outcomes:
    - ▪ Other single clinical assessments of uveitis activity
      • Anterior chamber cells (by SUN grade [4])
      • Presence of active inflammatory retinovascular lesion(s)
      • Presence of active inflammatory chorioretinal lesion(s)
      • Central macular thickness
    - ▪ Composite measure of uveitis activity
      • Time to treatment failure
    - ▪ Best corrected visual acuity (BCVA)
    - ▪ Heath-related quality of life
    - ▪ Concurrent requirement of corticosteroids to control uveitis
    - ▪ Concurrent requirement of other immunomodulatory treatment to control uveitis
    - ▪ Adverse events

Vitreous haze was chosen as the primary outcome, as firstly it has been identified by the FDA and other regulatory authorities as being a preferred trial endpoint for disease activity in uveitis of the posterior segment of the eye, and secondly, it is one of the commonest endpoints used in such trials [12]. Furthermore, a scoping search of mTOR inhibitor studies identified this as a common endpoint in these trials. We recognise however that vitreous haze is of limited intuitive value to patients, for whom an improvement in best corrected visual acuity or health-related quality of life may be more meaningful. These will be analysed as secondary outcomes in our systematic review, but it is widely acknowledged that neither are sufficiently sensitive over normal trial timescales to be used as the endpoint in clinical trials of uveitis, due to the indirect nature of disease activity to visual outcome [13].

### Selection process

The study selection process will be conducted in two stages:

- Title and abstract of the articles identified by the search strategy will be screened in order to remove irrelevant records.
- The full text of potentially relevant articles will be retrieved and assessed against the selection criteria above

Two reviewers will independently assess articles at both stages. Any disagreements will be resolved by discussion and if required referral to a third reviewer. Both stages of the selection process will be piloted and if necessary modified. The selection process will be illustrated using a PRISMA flow diagram, and details of articles excluded in the second (full text) stage will be recorded, along with the reason for exclusion [14].

Articles of non-English language will be translated (in part or wholly) to aid study selection and analysis.

### Data extraction

Two reviewers will independently extract data from the included articles. Any differences will be resolved by discussion, and if required referral to a third reviewer. A standardised, piloted data extraction form will be used. Study authors and publishing bodies may be contacted if further information is required.

For each study, the following information (but not limited to) will be extracted:

- Study characteristics
  - ○ Authors, publication year, title, journal, study design, setting, sample size, length of follow up, and analysis.
- Participant characteristics
  - ○ Patient selection and recruitment criteria, patient characteristics (demographic data, number, age, gender, ethnicity, socioeconomic status), diagnosis of non-infectious uveitis (including but not limited to: idiopathic, sarcoidosis, birdshot chorioretinopathy, Behcet’s disease), comorbidities, co-medication, information regarding prior uveitis treatment.
- Intervention and comparator
  - ○ Pharmacological agents studied, regimen (dose, frequency of administration, route of administration), comparator details (where present), differences in underlying care between treatment groups.
- Outcomes and findings
  - ○ Outcomes being measured and results for each outcome, precision and statistical test results for each outcome, completeness of follow-up for each outcome.

### Quality assessment

Two reviewers will independently assess the quality of the included articles. Any differences will be resolved by discussion, and if required referral to a third reviewer.

The Cochrane Handbook Risk of Bias tool will be used for RCTs [15].

Non-randomised controlled trials will also be assessed using the risk of bias tool for RCTs, accepting that criteria for randomisation and allocation concealment are not relevant.

The guidelines in Chapter 13 of the Cochrane Handbook will be followed for prospective controlled observational studies [15], however a minimum assessment can be made using the risk of bias tool for RCTs, again accepting than not all criteria may be relevant. The most relevant criteria to assess in these studies would relate to how groups were selected, differences in participant characteristics, losses to follow-up, biases and confounding in outcome assessment. The new Risk Of Bias In Non-randomized Studies - of Interventions (ROBINS-I) tool will also be used as a pilot [16].

The Newcastle-Ottawa Scale will be used to assess any case-controlled studies [17].

Case series will be assessed according to the guidance of the Centre for Reviews and Dissemination (York) [18].

### Analysis

Studies will be grouped by the intervention, comparator (if any) and design. Data will be tabulated and a narrative synthesis of evidence will be conducted for each outcome of relevance to the review and supported with a meta-analysis where appropriate.

Results for study outcomes may be presented using a variety of different measures, in continuous, discrete or dichotomous forms, within the same study or between studies.

For example, visual acuity may be reported in meters or feet from Snellen charts, LogMAR scores, or letters or lines read from ETDRS charts. Changes in acuity may be presented as any of these, or as a proportion passing over a threshold. Data conversion between formats for further analysis will be considered; however, any conversion will be done with due caution and regard to known issues, and the use of data conversion will be explicitly stated [19].

Regarding vitreous haze, which is commonly scored in clinical practice and research with the SUN/Nussenblatt scoring system, we are aware of a modification to this tool in occasional use, comprising an additional step to the established five-step scale. The additional step does not affect the value of the other Nussenblatt grading scores. This will preclude the combination of such studies with others where proportion of participants with a fixed step change (e.g. a proportion of participants with a two-step change) is the outcome metric, but not where the proportion having a fixed score is the metric (e.g. no haze) as the latter is the same on the original and modified scales.

Should multiple time-point data be available, either within the same study or between studies, data will be categorised for analysis by the post-intervention follow-up period. This will use groups of ≤ 3 months, > 3 and ≤ 6 months and > 6 months. Where appropriate, the > 6-month category may be further split up if long-term data is available.

For each time period for each outcome, studies contributing data will be assessed for, and where possible grouped according to, clinical and methodological homogeneity. This will determine the feasibility for undertaking meta-analysis and if undertaken whether a random effects or fixed effect model is most appropriate [20]. Data from differing study designs will not be pooled.

The *I*^2^ statistic (the percentage of data variability due to study heterogeneity) will be reported for each analysis undertaken. If a meta-analysis comprising 10 or more studies is undertaken, the possibility of publication bias will be investigated and a funnel plot constructed [21].

Where multiple studies report comparable continuous data, data using the same scale may be pooled using reported mean differences. Data on different scales may be pooled to derive a standardised mean difference. Subgroup analysis will be considered if appropriate.

If head-to-head studies comparing different mTOR agents exist, direct comparison of interventions will be undertaken. If multiple randomised controlled trials encompassing different mTOR agents and with differing comparators are found, the potential for undertaking indirect treatment comparisons will be considered to estimate a relative effect of the agents. Such comparison will be dependent on the studies available, and key assumptions regarding homogeneity, similarity and consistency of the studies [22, 23].

As the intervention under review comprises two agents (sirolimus and everolimus), evidence of the efficacy of the overall mTOR inhibitor class will be discussed to report on the consistency and magnitude of the overall class effect. Where available, comparison between the mTOR inhibitor class and other classes of agent will also be considered.

It is acknowledged that the number of studies included in this review is likely to be small and thus many of the analyses outlined above will not at the moment be possible. However, these methods are reported as this protocol is intended to inform future updates to this review where newer evidence may allow for these analyses.

### Reporting

This review and its findings will be reported in accordance with PRISMA guidelines [14]. The strengths and weaknesses of review methodology and evidence available will be discussed in relation to the external and internal validity of the findings. The review findings will be discussed in the context of current and future clinical practice regarding non-infectious uveitis, and possible future research in this area.

## Discussion and potential impact

Non-infectious uveitis is a potentially blinding inflammatory condition which often requires the prolonged use of high doses of corticosteroids. This can result in a significant burden of systemic steroid side effects, and there is an increasing clinical need for effective second-line therapeutics in order to achieve uveitis control, whilst reducing the need for excessive steroid doses (so-called steroid-sparing therapies). Various immunomodulatory therapies can be used as second-line agents for uveitis; however, these agents are often limited by a slower onset of action and reduced efficacy compared to corticosteroids (reducing their steroid-sparing potential), whilst being associated with different but equally detrimental side effects.

There is a need to consider the evidence for alternative second-line therapeutic agents with more favourable characteristics, and the mTOR inhibitors appear to be contenders for this role.

Second-line therapeutic agents are often introduced into ophthalmology after showing efficacy for inflammatory disease in other medical specialties, notably transplant immunosuppression and rheumatology [24]. Such agents are frequently used for years in an ‘off-label’ capacity in ophthalmology, based on clinical anecdote or personal experience, and few agents have high-level evidence to support their efficacy in uveitis [24]. This has led to considerable variability in the decision making process for second-line agents in uveitis.

Challenges in the attainment of high-level evidence for uveitis include the low incidence and prevalence of uveitis in the general population [5], and a heterogeneous aetiological basis with a historically inconsistent classification of uveitis phenotypes [24].

These challenges are gradually being addressed: the Standardisation of Uveitis Nomenclature group has worked to establish unified descriptions for uveitis which will aid in clinical practice, as well as collaboration and analysis across treatment centres [4]. Furthermore, larger, randomised and even multi-centre trials are becoming more common to evaluate new agents.

The mTOR inhibitors represent a small class of agents that, until recently, have been relatively neglected in ophthalmology. Like many second-line uveitis agents, they have a background in transplant immunosuppression; however, modern ophthalmic interest can be demonstrated by the imminent reporting of an international multicentre randomised controlled trial of sirolimus for non-infectious uveitis.

Therefore, it is timely to undertake a systematic review to examine the evidence for the use of mTOR inhibitors in non-infectious uveitis. Consequently, this review will clarify the available evidence for ophthalmologists and clinical decision makers regarding the potential role of mTOR inhibition in uveitis treatment protocols, in terms of agent efficacy, adverse event rates and the capability for corticosteroid dose reduction.

## Additional file

Additional file 1: PRISMA-P 2015 Checklist. (DOCX 25 kb)

## Appendix 1

**Table 1:** MEDLINE sample search strategy

Search number	Search details
1	Exp Uveitis/
2	(Uveitis OR Uveitic) ti, ab.
3	1 or 2
4	(Sirolimus OR rapamycin OR Rapamune OR 53123–88-9) ti. ab.
5	(Everolimus OR Afinitor OR Certican OR RAD 001 OR RAD001 OR SDZ RAD) ti. ab.
6	(Deforilimus OR Ridoforilimus OR AP23573 OR MK-8669) ti. ab.
7	(Temsirolimus OR CCI-779) ti. ab.
8	Exp mTOR inhibitor/
9	4 or 5 or 6 or 7 or 8
10	3 and 9

## Abbreviations

BCVA: Best corrected visual acuity
CENTRAL: Cochrane Central Register of Controlled Trials
CINAHL: Cumulative Index to Nursing and Allied Health Literature
EMBASE: Excerpta Medica Database
ETDRS: Early Treatment Diabetic Retinopathy Study
LogMAR: Logarithm of the Minimum Angle of Resolution
MEDLINE: Medical Literature Analysis and Retrieval System Online
mTOR: Mammalian target of rapamycin
PRISMA: Preferred Reporting Items for Systematic Reviews and Meta-Analyses
PROSPERO: International prospective register of systematic reviews
RCT: Randomised controlled trial
WHO: World Health Organization
ZETOC: Z39.50-compliant access to the British Library Electronic Table of Contents
